# Co-infection in patients with hypoxemic pneumonia due to COVID-19 in Reunion Island

**DOI:** 10.1097/MD.0000000000024524

**Published:** 2021-01-29

**Authors:** Nicolas Allou, Kevin Larsen, Arthur Dubernet, Nicolas Traversier, Laurie Masse, Emilie Foch, Léa Bruneau, Adrien Maillot, Michel André, Marie Lagrange-Xelot, Jérôme Allyn, Vincent Thomas, Nathalie Coolen-Allou

**Affiliations:** a Réanimation polyvalente; b Département d’Informatique Clinique; c Pneumologie; d Médecine Interne; e Microbiologie, Centre Hospitalier Universitaire Felix Guyon Allée des Topazes, Saint Denis; f INSERM CIC 1410 Clinical and Epidemiology, University Hospital, Saint Pierre; g Department of Public health and research support, Methodological Support and Biostatistics Unit, University Hospital, Saint Denis, Reunion Island; h Service des Maladies Infectieuses, Centre Hospitalier Universitaire Felix Guyon Allée des Topazes, Saint Denis, France.

**Keywords:** co-infection, coronavirus disease 2019, influenza, pneumonia, SARS-CoV-2, *Staphylococcus aureus*

## Abstract

This study aimed to evaluate the incidence of co-infection with different types of pathogens in patients with hypoxemic pneumonia due to coronavirus disease 2019 (COVID-19) in Reunion Island.

This observational study using a prospectively collected database of hypoxemic pneumonia due to COVID-19 cases was conducted at Félix Guyon University Hospital in Reunion Island, France.

Between 18 March 2020 and 15 April 2020, 156 patients were admitted to our hospital for COVID-19. A total of 36 patients had hypoxemic pneumonia (23.1%) due to COVID-19. Thirty of these cases (83.3%) were imported by travelers returning mainly from metropolitan France and Spain. Patients were screened for co-infection with other pathogens at admission: 31 (86.1%) by multiplex polymerase chain reaction (PCR) and 16 (44.4%) by cytobacteriological examination of sputum culture. Five patients (13.9%) were found to have co-infection: 1 with influenza virus A H1N1 (pdm09) associated with *Branhamella catarrhalis*, 1 with *Streptococcus pneumoniae* associated with *Haemophilus influenzae*, 1 with Human Coronavirus 229E, 1 with Rhinovirus, and 1 with methicillin-susceptible *Staphylococcus aureus*. Patients with co-infection had higher D-dimer levels than those without co-infection (1.36 [1.34–2.36] μg/mL vs 0.63 [0.51–1.12] μg/mL, *P* = .05).

The incidence of co-infection in our cohort was higher than expected (13.9%). Three co-infections (with influenza virus A(H1N1) pdm09, *Streptococcus pneumoniae*, and *Staphylococcus aureus*) required specific treatment. Patients with hypoxemic pneumonia due to COVID-19 should be screened for co-infection using respiratory cultures or multiplex PCR. Whilst our study has a number of limitations, the results from our study suggest that in the absence of screening, patients should be commenced on treatment for co-infection in the presence of an elevated D-dimer.

## 1 Introduction

Coronavirus disease 2019 (COVID-19) first emerged in China in December 2019.^[1]^ Since then, the disease has spread rapidly across all continents. The COVID-19 pandemic is overwhelmingly associated with international travel.^[2–5]^ As of 29 September 2020, there were nearly 34 million cases of COVID-19 infection worldwide, with more than 1 million deaths.^[6]^

Reunion Island, a French overseas department located in the Indian Ocean region, is connected to Paris, South Africa, India, China, and Thailand through several daily flights. On 29 September 2020, 3882 cases of COVID-19 had been reported on Reunion Island (first case detected on 11 March 2011). During the first epidemic period (March to July 2020), 667 cases of COVID-19 had been reported. The vast majority of these cases were imported from Europe (France, Spain, and Italy) and Comoros Archipelago.^[7]^

To date, studies that explored cases of co-infection between COVID-19 and other microorganisms were mainly carried out in Asia.^[8–13]^ The aim of our study was to evaluate the incidence of co-infection with different types of pathogens in patients with hypoxemic pneumonia due to COVID-19 in Reunion Island during the first epidemic period.

## 2 Methods

The present observational study was approved by the Ethics Committee of the French Society of Pulmonary Medicine and was declared to the Commission nationale de l’informatique et des libertés (French Data Protection N°2206739). The need for informed consent was waived, as the study was non-interventional and followed our usual protocol. However, all patients or their legally authorised representative were verbally informed about the process of data collection and could refuse to participate in the study.

This study complies with the Strengthening the Reporting of Observational studies in Epidemiology recommendations statement.^[14]^

### 2.1 Selection of the study sample

This retrospective observational cohort study using a prospectively collected database was conducted between 18 March 2020 and 15 April 2020 at Félix Guyon University Hospital, the only hospital authorized to manage patients with COVID-19 in Reunion Island, France. All patients diagnosed with COVID-19 in another hospital of the Island were systematically transferred to our hospital.

During this study, hospitalization policy has changed according to the guidelines from the French Ministry of Health, depending on the stage of the outbreak. From 11 to March 24, 2020, all diagnosed cases were systematically hospitalized for 14 days. As of March 25, 2020 (start of the spread of COVID-19 in Reunion Island), only cases with severity's signs were hospitalized.

All consecutive patients with COVID-19 confirmed by polymerase chain reaction (PCR) and presenting with hypoxemic pneumonia were evaluated.

All computed tomography images were analyzed by at least 2 pulmonologists (LM, EF, MA, NCA) blinded to clinical information. Six stages of severity of pulmonary involvement were defined based on these images:

(1) none;
(2) low (<10%);
(3) moderate (10% to 25%);
(4) extended (25% to 50%);
(5) severe (50% to 75%);
(6) critical (>75%).^[15]^

Hypoxemic pneumonia was defined as pneumonia requiring oxygen supplementation to achieve oxyhemoglobin saturation > 94%.

All patients with hypoxemic pneumonia due to COVID-19 were treated with a 3rd generation cephalosporin not active against *Pseudomonas aeruginosa*.

In the absence of contraindications, patients with symptoms for less than 10 days were treated with oral hydroxychloroquine for a period of 10 days, in association with azithromycin for a period of 5 days.^[17,18]^

### 2.2 Microbiological investigations

Samples used (nasopharyngeal swab in non-intubated and tracheal aspirate in intubated patients) for COVID-19 screening were tested by Multiplex PCR (Seegene Allplex respiratory panel, eurobio ingen, Les Ulis, France) for the following pathogens: Influenza, Respiratory Syncytial Virus, Adenovirus, Enterovirus, Parainfluenza, Human Metapneumovirus, Human Bocavirus, Rhinovirus, Coronavirus (NL63, 229E and OC43), *Chlamydia pneumoniae*, *Mycoplasma pneumoniae*, *Legionella spp*, *Haemophilus influenza*, *Streptococcus pneumoniae* and *Bordetella (para) pertussis.*

Pneumococcal and Legionella urinary antigen tests, cytobacteriological examination of sputum cultures, and serology of atypical respiratory pathogens were performed at the physician's discretion (*DiaSorin-LIAISON XL (CLIA) for* Mycoplasma pneumoniae *and* NovaLISA/NovaTec technique (Biomnis, EIA) for *Chlamydia pneumoniae*).

### 2.3 Data collection and study outcome

Patient comorbidities at hospital admission were recorded. Clinical and biological data were collected at the time of diagnosis of hypoxemic pneumonia due to COVID-19.

The primary outcome was to identify the microorganisms responsible for co-infection in patients with hypoxemic pneumonia due to COVID-19.

The secondary outcome was to evaluate the clinical, biological, and computed tomography scan characteristics of patients with COVID-19 pneumonia complicated by co-infection.

### 2.4 Statistical analysis

Results were expressed as total numbers (percentages) for categorical variables and as medians [25th–75th percentiles] for continuous variables. Categorical variables were compared using the Chi-square test or the Fisher exact test, as appropriate. A *P-*value < .05 was considered significant. Analyses were performed using SAS statistical software (8.2, Cary, NC, USA).

## 3 Results

### 3.1 Population

In summary, 156 patients were admitted to our hospital for COVID-19 over the study period. Among the 156 patients median age was 50 (33–62) years old, 83 were male gender (53%), 36 had hypertension (23%), 47 had body mass index > 25 kg/m^2^ (30%) and 22 had diabetes mellitus (14%). A total of 36 patients were found to have hypoxemic pneumonia (23.1%). Characteristics of patient with hypoxemic pneumonia are described in Tables 1 and 2. Of these 36 patients, 30 patients had recently returned from one of the countries most affected by the COVID-19 outbreak (83.3%): 26 from metropolitan France, 6 from Spain, 2 from Italy, 1 from the United States, and 1 from the United Kingdom (some patients had visited several of these countries). Among the 36 patients with hypoxemic pneumonia, the median age was 65.5 [56–77] years, 10 were hospitalized in intensive care and the median time from symptom onset to diagnosis of COVID-19 infection was 5.5 [2.5–7] days.

**Table 1:** Demographic characteristics and comorbidities at hospital admission of the 36 patients with hypoxemic pneumonia due to COVID-19.

		Co-infection		
Characteristics	Total (n = 36)	No (n = 31)	Yes (n = 5)	*P*-value
Male sex, n (%)	25 (69.4)	21 (67.7)	4 (80)	.51
Age, median [25th-75th], years old	66 [56–77]	66 [57–74]	68 [57–82]	.723
Hypertension, n (%)	12 (33.3)	10 (32.3)	2 (40)	.55
Diabetes mellitus, n (%)	5 (13.9)	5 (16.1)	0	.45
Dyslipidemia, n (%)	6 (16.7)	6 (19.4)	0	.29
Body mass index > 30 kg/m^2^, n (%)	6 (16.7)	5 (16.1)	1 (20)	.62
Body mass index, median [25th-75th], kg/m^2^	26 [23.2–27.5]	26 [23–27]	27 [25–30.5]	.3
Chronic kidney disease requiring hemodialysis	3 (8.3)	3 (9.7)	0	.63
Chronic obstructive pulmonary disease, n (%)	10 (27.8)	8 (25.8)	2 (40)	.43
History of congestive heart failure (NYHA class ≥ III), n (%)	8 (22.2)	7 (22.6)	1 (20)	.69
Cancer (< 3 months), n (%)	2 (5.6)	1 (3.2)	1 (20)	26
History of stroke, n (%)	3 (8.3)	1 (3.2)	2 (40)	.05
^∗^Immunodepression, n (%)	1 (2.8)	1 (3.2)	0	.86
Tobacco smoking (current or former), n (%)	11 (30.6)	9 (29)	2 (40)	.76
^†^Renin-Angiotensin System Inhibitor therapy, n (%)	8 (22.2)	7 (22.6)	1 (20)	.68
Statin therapy, n (%)	7 (19.4)	6 (19.4)	1 (20)	.55

Results are expressed at n (%) or median [25th-75th] as appropriate. NYHA = New York Heart Association.

∗ included any immunosuppressive diseases, such as congenital or acquired immunodeficiency, hematologic diseases, treatment with immunosuppressive drugs within the previous 30 days, or corticosteroids in daily doses of at least 10 mg/day of a prednisone equivalent for more than 2 weeks,.

† angiotensin-converting enzyme or angiotensin II receptor blockers.

**Table 2:** Clinical and biological characteristics at hospital admission of the 36 patients with hypoxemic pneumonia due to COVID-19.

		Co-infection		
Characteristics	Total (n = 36)	No (n = 31)	Yes (n = 5)	*P*-value
Delay between diagnosis and onset of symptoms, median [25^th^–75th], days	5.5 [2.5–7]	5 [4–7]	6 [2–6]	.79
Body core temperature, median [25th–75th], °C	37.5 [37–38.7]	37.5 [37.2–38.6]	37.5 [36.7–38.5]	.79
Respiratory rate, median [25th–75th], breaths per min	24 [20–26]	22 [19–25]	27 [25–29]	.07
Herat rate, median [25th–75th], beats per min	85 [72–101]	81 [76–101]	89 [88–97]	.79
Oxyhemoglobin saturation, median [25th–75th], %	95 [94–97]	95 [95–97]	94 [92–96]	.35
Oxygen therapy, median [25th–75th], L/min	3 [1.8–4]	3 [2–4]	2 [1–3]	.56
Influenza-like illness, n (%)	15 (41.7)	14 (45.2)	1 (20)	.29
Asthenia, n (%)	14 (38.9)	14 (45.2)	0	.07
Cough, n (%)	24 (66.7)	21 (67.7)	3 (60)	.55
Chest pain, n (%)	4 (11.1)	4 (12.9)	0	.53
^∗^Acute respiatory failure, n (%)	5 (13.9)	4 (12.9)	1 (20)	.55
Dyspnea, n (%)	18 (50)	16 (51.6)	2 (40)	.5
Digestive disorders, n (%)	10 (27.8)	10 (32.3)	0	.17
Headache, n (%)	7 (19.4)	7 (22.6)	0	.32
Olfactory and or gustatory dysfunctions, n (%)	7 (19.4)	7 (22.6)	0	.32
Confusion, n (%)	2 (5.6)	2 (6.5)	0	.74
Leucocyte, median [25th–75th], G/L	5.6 [4.2–9.8]	5.22 [4.09–8.39]	10.4 [6.8–13.66]	.07
Polynuclear neutrophils, median [25th–75th], G/L	3.3 [2.3–6.6]	3.32 [2.65–5.77]	7.36 [5.4–10.86]	.15
Lymphocytes count, median [25th–75th], G/L	1.2 [0.85–1.49]	1.16 [0.81–1.415]	1.5 [1.22–1.93]	.16
Polynuclear neutrophils/Lymphocytes ratio	3.3 [2.3–6.6]	3.1 [2.3–6.1]	5.9 [3.8–7.2]	.42
Eosinophils, median [25th–75th], G/L	0.02 [0–0.13]	0.07 [0.01–0.09]	0.07 [0.01–0.09]	.825
Platelet count, median [25th–75th], G/L	182 [155–300]	181 [157–313]	264 [141–265]	.86
Hemoglobin level, median [25th–75th], g/dL	12.9 [12–13.8]	12.9 [11.9–13.8]	13.1 [12.8–13.4]	.42
D-dimer level, median [25th–75th], μg/mL	0.83 [0.51–1.33]	0.63 [0.51–1.12]	1.36 [1.34–2.36]	.05
Prothrombin time, median [25th–75th], %	79 [68–93]	79 [69–93]	73 [57–87]	.51
C-reactive protein, median [25th–75th], mg/dL	73.6 [18.3–113.1]	75.7 [23.5–111.4]	43 [14–87]	.6
Cardiac troponin I > 10 ng/L	8 (22.2)	7 (22.6)	1 (25)	.69
Bilirubin level, median [25^th^–75th], mg/dL	10.5 [9–13.8]	10 [9–12]	16 [11–19.5]	.29
Serum albumin, median [25th–75th], g/L	26 [24.5–29.5]	26 [24–30]	28.3 [28.3–28.9]	.48
paO2, median [25th–75th], mmHg	83 [76–105]	83 [76–104]	99 [88–111]	.62
paCO2, median [25th–75th], mmHg	36 [33–40]	36 [33–40]	36 [34–40]	.89
Lactate dehydrogenase, median [25th–75th], IU/L	376 [285–429]	376 [287–429]	323 [286–361]	.63
Aspartate aminotransferase level, median [25th–75th], U/L	58 [36–76]	56 [38–68]	71 [57–87]	.43
Creatinine, median [25th–75th], μmol/L	87 [66–95]	86 [70.4–95]	93 [64–105]	.48

Results are expressed at n (%) or median [25th–75th] as appropriate. PaCO_2_ = Arterial partial pressure of carbon dioxide, PaO_2_ = Arterial oxygen partial.

∗ invasive mechanical ventilation or high-flow nasal cannula oxygen therapy.

Five patients (13.9%) were found to have co-infection. Multiplex PCR was performed on samples used to screen for COVID-19 infection in 31 out of 36 patients (86.1%) with hypoxemic pneumonia due to COVID-19. Four patients were found to be positive for other microorganisms: 1 for influenza virus A(H1N1) pdm09, 1 for *Streptococcus pneumonia* associated with *Haemophilus influenzae*, 1 for human coronavirus 229E, and 1 for rhinovirus.

Cytobacteriological examination of the sputum sample was performed in 16 patients. One patient tested positive for methicillin-susceptible *Staphylococcus aureus*, and another tested positive for *Branhamella catarrhalis* (this patient had a co-infection with the influenza virus A H1N1).

Lastly, 15 pneumococcal and legionella urinary antigen tests, 24 serologies for *Mycoplasma pneumoniae* and *Chlamydia pneumoniae* were performed. All these tests gave negative results.

### 3.2 Factors predictive of co-infection and outcome

In univariate analysis, no differences in clinical parameters were found between patients with and without co-infection (Tables 1 and 2). The only biomarker significantly associated with co-infection was D-dimer levels (1.36 [1.34–2.36] μg/mL in patients with co-infection vs. 0.63 [0.51–1.12] μg/mL in patients without co-infection, *P* = .05). No difference in disease severity was observed on chest computed tomography scan between patients with and without co-infection (Table 3). In-hospital length of stay was similar between the two groups of patients: 10 [8–16] days in the group with co-infection and 13 [10–19] days in the group without co-infection (*P* = .3).

**Table 3:** Chest computed tomography scan characteristics of 34 patients with hypoxemic pneumonia due to COVID-19.

		Co-infection		
Characteristics	Total (n = 34)	No (n = 30)	Yes (n = 4)	*P* value
Time from onset of symptoms				
Pulmonary infiltrates, n (%)	34 (100)	30 (100)	4 (100)	.96
Bilateral involvment, n (%)	33 (97.1)	29 (96.7)	4 (100)	.99
Extension of pulmonary infiltrates:				.58
-stage 0: none, n (%)	0	0	0	
- stage 1: low (<10%), n (%)	0	0	0	
- stage 2: moderate (10 to 25%), n (%)	18 (52.9)	16 (53.3)	2 (50)	
- stage 3: extended (25 to 50%), n (%)	7 (20.6)	6 (20)	1 (25)	
- stage 4: severe (50 to 75%), n (%)	9 (26.5)	8 (26.7)	1 (25)	
- stage 5: critical (>75%), n (%)	0	0	0	
Ground glass opacities, n (%)	33 (97.1)	29 (96.7)	4 (100)	.97
Consolidations, n (%)	27 (79.4)	24 (80)	3 (75)	.62
Crazy paving, n (%)	19 (55.9)	17 (56.7)	2 (50)	.6
Pleural effusion, n (%)	6 (17.6)	5 (16.7)	1 (25)	.56

Results are expressed at n (%) or median [25th–75th]. Thirty-four of the 36 patients with COVID-19 pneumonia underwent a chest computed tomography scan.

Four patients (10.5%) (all of whom were hospitalized in intensive care unit) developed a nosocomial infection: 2 developed central venous catheter-related infection due to coagulase-negative Staphylococci (1 of which was associated with bacteremia), 2 developed bacteremia secondary to ventilator-associated pneumonia due to *Pseudomonas aeruginosa* in the first case and to *Burkholderia cepacia* in the second case. Median in-hospital length of stay was not significantly different between the 2 groups of patients: 26 [19–31] days in the group with nosocomial infection and 12 [10–15] days in the group without nosocomial infection (*P* = .09). Ten of the 36 patients with hypoxemic pneumonia were admitted to ICU (27.8%). Of these, 3 patients received high-flow nasal cannula oxygen therapy and 2 received invasive mechanical ventilation. Among the 36 patients, there were no deaths at follow-up. At the time of writing, all hospitalized patients were discharged from hospital.

## 4 Discussion

The incidence of co-infection in our patients with hypoxemic pneumonia in Reunion Island was higher than expected (13.9%). In the published literature, the incidence of co-infection in patients with COVID-19 pneumonia is highly variable, ranging from 0% to 20%.^[8–13]^ To date, studies that explored cases of co-infection between COVID-19 and other microorganisms were mainly carried out in Asia.^[8–13]^

It appears that the incidence of co-infection was higher in studies that evaluated intensive care patients.^[9,10]^ The different microorganisms isolated were very different according to the studies with a high proportion of *Mycoplasma pneumoniae* in some studies and especially in China.^[9–13]^ The Influenza virus was also one of the most common pathogens responsible for co-infection.^[9,11]^ It should be noted that these microorganisms are treatable and known to be true pathogens of the upper airways.

In our study, 2 different bacteria (1 strain of *Staphylococcus aureus* and 1 strain of *Branhamella catarrhalis*) were isolated in sputum samples taken from 2 patients. This is unsurprising, as viruses, and especially the influenza virus, are known to be frequently associated with bacteria such as *Staphylococcus aureus*.^[16]^

The only biomarker to be associated with co-infection in our cohort was D-dimer levels. Several studies have found D-dimer levels to be a prognostic marker of severity in COVID-19 infection.^[17]^ In view of this, patients with severe COVID-19 infection and high D-dimer levels should be treated without delay, ideally with an antibiotic not active against *Pseudomonas aeruginosa* (e.g., 3rd generation cephalosporin or amoxicillin/clavulanate). Alternatively, oseltamivir treatment may be administered to patients at risk of developing co-infection with the Influenza virus.

Azithromycin may be an interesting molecule for the treatment of COVID-19 patients with co-infection. Indeed, azithromycin has a broad antibacterial spectrum, which is active against atypical germs like *Mycoplasma pneumoniae*.^[18]^ Moreover, it has well-known anti-inflammatory activity and may be effective against COVID-19 even in the absence of pyogenic infection.^[18]^ It should be noted, however, that the hydroxychloroquine/azithromycin treatment was not associated with better outcome in some studies.^[19,20]^

Our study has many limitations that must be acknowledged. First, our cohort was relatively small. Another limitation of our study is that our microbiological results are difficult to extrapolate because of the variable distribution of infective microorganisms across geographical areas. While the co-infections analyzed in our study were diagnosed in the southern hemisphere, the vast majority of our patients had recently traveled to the northern hemisphere, making it almost impossible to trace the source of their co-infection. It is indeed difficult to determine whether a patient co-infected with Influenza caught the virus in Europe, where Influenza season in nearing its end, or in Reunion Island, where the virus is present all year round with a peak during the southern winter (May to October).^[21,22]^ Only data concerning patients with hypoxemic pneumonia were collected. However, it is not recommended to initiate an anti-infective treatment only in a patient without severe pneumonia due to COVID-19 (i.e. hypoxemic pneumonia).^[23]^ It is difficult to ascertain whether the infectious agents are actually implicated in disease, rather than occupying an ecological niche in the nasopharynx or the lung without causing disease. Nevertheless, in clinical practice, it is difficult not to treat the presence of some microorganisms as *Staphylococcus spp* or Influenza in a patient with severe pneumonia. Lastly, few of our patients underwent all the microbiological diagnostic tests (sputum cultures, serologies, antigen tests). Cytobacteriological examination would have likely been more sensitive had we used deep respiratory samples instead of sputum samples. However, we preferred not to subject our patients to invasive procedures when not entirely necessary, especially since the vast majority of them did not require invasive mechanical ventilation.

Likewise, it may be that multiplex PCR would have performed better on deep respiratory samples. Yet, studies comparing the diagnostic performance of PCR on nasopharyngeal vs. deep respiratory samples have found no major difference between the 2 techniques, except in the case of some microorganisms such as Legionella pneumophilia.^[24]^

## 5 Conclusion

The incidence of co-infection in our patients with hypoxemia pneumonia due to COVID-19 was higher than expected (13.9%). COVID-19 patients who present with hypoxemic pneumonia should be be screened for co-infection using respiratory cultures or multiplex PCR in order to isolate treatable pathogens like influenza virus or pyogenic germs. Whilst our study has a number of limitations, the results from our study suggest that in the absence of screening, patients should be commenced on treatment for co-infection in the presence of an elevated D-dimer. Larger studies should be conducted to determine the clinical, biological, and radiological characteristics that indicate the presence of co-infection in patients with COVID-19.

## Author contributions

**Conceptualization:** Nicolas Allou, Kevin Larsen, Arthur Dubernet, Laurie Masse, Emilie Foch, Adrien Maillot, Michel Andre, Marie Lagrange-Xelot, Vincent Thomas.

**Data curation:** Kevin Larsen, Emilie Foch, Lea Bruneau, Marie Lagrange-Xelot, Jérôme Allyn, Vincent Thomas, Nathalie Coolen-Allou.

**Formal analysis:** Nicolas Allou, Arthur Dubernet, Nicolas Traversier, Laurie Masse, Adrien Maillot, Michel Andre, Jérôme Allyn, Nathalie Coolen-Allou.

**Funding acquisition:** Marie Lagrange-Xelot.

**Investigation:** Nicolas Allou, Arthur Dubernet, Nicolas Traversier, Laurie Masse, Emilie Foch, Lea Bruneau, Michel Andre, Marie Lagrange-Xelot, Jérôme Allyn, Nathalie Coolen-Allou.

**Methodology:** Nicolas Allou, Kevin Larsen, Arthur Dubernet, Laurie Masse, Emilie Foch, Lea Bruneau, Adrien Maillot, Michel Andre, Marie Lagrange-Xelot, Jérôme Allyn, Vincent Thomas, Nathalie Coolen-Allou.

**Supervision:** Nicolas Allou, Kevin Larsen, Marie Lagrange-Xelot, Vincent Thomas.

**Validation:** Nicolas Allou, Kevin Larsen, Arthur Dubernet, Nicolas Traversier, Emilie Foch, Marie Lagrange-Xelot, Jérôme Allyn, Vincent Thomas, Nathalie Coolen-Allou.

**Visualization:** Nicolas Allou, Arthur Dubernet, Nicolas Traversier, Nathalie Coolen-Allou.

**Writing – original draft:** Nicolas Allou, Kevin Larsen, Arthur Dubernet, Emilie Foch.

**Writing – review & editing:** Nicolas Allou.

## Abbreviations

Abbreviations: COVID-19 = coronavirus disease 2019, PCR = polymerase chain reaction.
